# Using Urinary Biomarkers to Estimate the Benzene Exposure Levels in Individuals Exposed to Benzene

**DOI:** 10.3390/toxics10110636

**Published:** 2022-10-23

**Authors:** Shiwei Cui, Bo Pang, Huifang Yan, Bo Wu, Ming Li, Caihong Xing, Juan Li

**Affiliations:** 1 Department of Hygienic Inspection, School of Public Health, Jilin University, Changchun 130021, China; 2 National Institute for Occupational Health and Poison Control, Chinese Center for Disease Control and Prevention, Beijing 100050, China; 3 Science and Technology Research Center of China Customs, Beijing 100026, China; 4 Department of Occupational Health, Jinan Railway Disease Control and Prevention Center, Jinan 250001, China

**Keywords:** benzene exposure, biological monitoring, daily intake, S-PMA, *t, t*-MA

## Abstract

Urinary benzene metabolites trans, trans-muconic acid (*t, t*-MA), and S-phenyl mercapturic acid (S-PMA) are often used as biomarkers of internal exposure to benzene. However, there are few reports on using urinary benzene metabolites to estimate airborne benzene concentrations in individuals exposed to benzene. In this study, *t, t*-MA, and S-PMA were analyzed by UPLC-MS/MS, and a simple pharmacokinetic model was used to calculate the daily intake (DI) of benzene based on the levels of urinary *t, t*-MA, and S-PMA in occupational individuals. The back-calculated airborne benzene levels (BCABL) were obtained from the DI of benzene. Among the exposed subjects (*n* = 84), the median BCABL (3.67 mg/m^3^) based on *t, t*-MA was very close to the median level of measured airborne benzene (3.27 mg/m^3^, *p* = 0.171), and there was no effect of smoking or dietary habits on *t, t*-MA-based BCABL. In the control subjects (*n* = 49), the levels of measured airborne benzene were all below the quantitation limit (0.024 mg/m^3^), and the BCABL (0.002–0.25 mg/m^3^) calculated by S-PMA was close to this background level. Our study suggests that the *t, t*-MA-based BCABL can reflect the actual airborne benzene level in a range of 1.10–86.91 mg/m^3^ and that the S-PMA-based BCABL is more reliable for non-professional benzene exposure.

## 1. Introduction

Benzene is one of the simplest aromatic hydrocarbons. Because of its excellent thermodynamic stability and solubility, it has been widely used as a chemical synthesis intermediate and an industrial solvent in the industry. Nowadays, occupational exposure to benzene in China primarily occurs in the manufacturing industry, such as furniture manufacturing, printing, and the recording media reproduction industry [1]. Benzene is also found in automobile exhausts, flue gas, and cigarette smoke [2]. Chronic exposure to benzene can cause acute myeloid leukemia [3,4,5] and is associated with other blood-forming system diseases, such as aplastic anemia, myelodysplastic syndrome (MDS), and lymphocytic leukemias [6,7,8]. It is classified as a Group I carcinogen by the International Agency for Research on Cancer (IARC) [9].

In the past few decades, benzene exposure levels in the workplace have gradually decreased. The median concentration of benzene is below 3 mg/m^3^ in more than 98% of benzene industries from six major benzene-exposed provinces in China [1]. The median concentration of benzene is 1.50 mg/m^3^ (the conversion scale between ppm and mg/m^3^ is 3.25 for benzene at 20 °C, the air concentration in this paper is displayed with mg/m^3^ or μg/m^3^) among petrochemical workers in Bulgarian [10] and is 32.6 μg/m^3^ among refinery workers in Southern Italy [11]. However, benzene is harmful to human health even at a very low exposure level, and there is no evidence of a threshold. In a cross-sectional study of 390 workers, white blood cell and platelet counts were significantly decreased in workers exposed to <3.25 mg/m^3^ of benzene [12]. Another study has shown that the levels of serum protein adducts increased in workers exposed to low concentrations of benzene (interquartile range: 6.96–43.6 mg/m^3^), which indicated that low concentrations of benzene exposure might increase the risk of leukemia [13]. 

In order to effectively assess the health effects of benzene exposure, it is crucial to determine the actual absorbed dose of the benzene-exposed population. After benzene enters the human body, it is metabolized to benzene oxide by cytochrome P450 (CYP450). As products of the further metabolism of benzene oxide, trans, trans muconic acid (*t, t*-MA), and S-phenyl mercapturic acid (S-PMA) have been applied as internal exposure biomarkers to assess the risk of benzene exposure. Previous studies found that the urinary concentration of *t, t*-MA and S-PMA were consistently elevated when benzene exposure was above 0.65 mg/m^3^ [14]. Additionally, both *t, t*-MA, and S-PMA positively correlated with airborne benzene among 0.2–396.5 mg/m^3^ [15]. The individual daily intake (DI) of pollutants can be calculated using a simplified pharmacokinetic model based on the concentration of metabolites in urine. This model has been used to estimate the DI of phthalates [16,17] and benzene [18] in non-occupational exposed individuals. Therefore, the pharmacokinetic model successfully linked airborne benzene exposure to urinary benzene metabolite concentrations and, as a result, potentially contributed to the risk assessment of occupational benzene exposure. 

This study aimed to use DI obtained by a simple pharmacokinetic model based on the levels of urinary *t, t*-MA, and S-PMA to back the calculated airborne benzene levels (BCABL) in individuals exposed to benzene. Under the factors of benzene exposure levels, smoking habits, and dietary habits, the correlation between airborne benzene levels and BCABL was analyzed. 

## 2. Materials and Method

### 2.1. Study Population

The study population included 133 subjects. A total of 84 subjects, who were employed at a shoe factory in Shandong Province, China, formed part of the exposed group, and another 49 unexposed subjects from a food factory in the same geographic area were the control group. The unexposed subjects were frequency-matched with the exposed workers for age and gender. The workers involved in the study were informed of the study’s aims, and each participant signed an informed consent form.

### 2.2. Personal Exposure

During the entire work shift of the subjects, benzene exposure was monitored using a personal diffusive sampler. Benzene was collected by active carbon particles (SKCINC^®^) filled in the sampler. Afterward, benzene was desorbed using carbon disulfide, then measured using gas chromatography with a flame ionization detector (GC-FID). The limit of the quantitation of the analysis procedure was 0.024 mg/m^3^.

### 2.3. Urine Analysis

A sterile plastic bottle was used to collect the urine of each worker at the end of their work shift. Each collected urine sample was stored at −40 °C before being divided into two parts, one at 10 mL for the determination of urinary creatinine and the other at 20 mL for the determination of S-PMA and *t, t*-MA using Ultra Performance LC (Waters, Milford, MA, USA) tandem mass spectrometry (AB SCIEX QTRAP 5500, Framingham, MA, USA) and analytical methods with isotopic dilution.

As briefly described below, the method by Paci et al. [19] was modified for the determination of S-PMA. Firstly, a 1 mL urine sample was treated with 10 μL of a deuterated internal standard solution in methanol (S-PMA-d5, 0.5 mg/L) and 0.3 mL 9 M H_2_SO_4_ for the hydrolysis of the urinary precursor pre-SPMA for 10 min, and then 0.25 mL NaOH 50% in water was added to adjust the pH. Then, the urine samples were purified with solid phase extraction columns (C18 ODS, sorbent bed mass 500 mg, Agilent, Santa Clara, CA, USA), and the pretreatment method was optimized. The C18 ODS cartridge was balanced and pretreated with 3 mL of methanol and 3 mL 0.1% acetic acid. Then, the urine hydrolysate was loaded into the C18 ODS cartridge. The analytes of interest were eluted with 1 mL of methanol after washing the C18 ODS cartridge with 3 mL of 0.1% of acetic acid. The eluate was filtered on a 0.2 μm filter device and 10 μL was injected into the UPLC-MS/MS system, using a linear gradient of 20% methanol and 80% 0.1 mol/L acetic acids, with up to 90% methanol and an ACQUITY UPLC BEH C_18_ analytical column (2.1 × 50 mm, 1.7 μm, Waters, Milford, MA, USA). The negative ions and multiple reaction ion monitoring (MRM) modes were used. The ionic transitions monitored were as follows: *m*/*z* 238.1–*m*/*z* 109.1 for S-PMA and *m*/*z* 243.0–*m*/*z* 114.1 for the deuterated internal standard. If the content of S-PMA in the urine samples exceeded the linear range, the eluent would be diluted by methanol before analysis.

For *t, t*-MA a 1 mL portion of each sample was treated with 0.33 mL of phosphate buffer (0.59 g of KH_2_PO_4_ and 2.15 g Na_2_HPO_4_ in 500 mL water, pH = 7.4) and 50 μL of a deuterated internal standard solution in methanol (*t, t*-MA-d4, 4 mg/L). Then, the urine samples were purified with Bond Elut SAX cartridges (sorbent bed mass 500 mg, Agilent, Santa Clara, CA, USA). Bond Elut SAX cartridges were previously conditioned with 3 mL of methanol and 3 mL of 0.1% (*v*/*v*) acetic acid in water, and after loading the samples, the cartridges were washed with 3 mL of 0.1% (*v*/*v*) acetic acid in water and 3 mL of methanol. Finally the analytes of interest were eluted with 3 mL 10% (*v*/*v*) acetic acid in water. The eluate was filtered on a 0.2 μm filter device, and 10 μL was injected into the UPLC-MS/MS system using a linear gradient of 10% acetonitrile and 90% 0.1% acetic acid, with up to 90% acetonitrile [20]. An ACQUITY UPLC BEH Shield RP18 column (2.1 × 100 mm, 1.7 μm, Waters, USA) were used for the analysis of *t, t*-MA. The following *m*/*z* ion combinations (precursor—product) were monitored and the transitions (negative mode) were as follows: 141.0–97.0 for *t, t*-MA and *m*/*z* 145.0–100.0 for the deuterated internal standard. If the content of *t, t*-MA in the urine samples exceeded the linear range, the eluent would be diluted by 10% (*v*/*v*) acetic acid in water before analyzing.

The linear range was 0.17–50 μg/L for S-PMA and 3.3–1000 μg/L for *t, t*-MA. The precision and accuracy at the level of 5.0 µg/L were 2.1% and 99.8% for S-PMA, and at the level of 25.0 µg/L, it was 1.6% and 102%. For *t, t*-MA, precision and accuracy are 5.6% and 101% at the level of 50 µg/L, and at the level of 200 µg/L, it is 3.4% and 98.3%. The limits of quantitation (LOQs) were 0.17 μg/L and 3.3 μg/L for S-PMA and *t, t*-MA, respectively. A validated HPLC-UV method was used to determine the urinary creatinine for each subject. The urine was diluted appropriately, and the peak was detected at 254 nm [21].

### 2.4. Calculation of Benzene Daily Intake (DI)

Based on the urinary metabolites and urinary creatinine levels, the values of S-PMA and *t, t*-MA were converted to show the value of benzene daily intake (DI) by applying the following Equation (1), which has already been used to calculate the daily phthalate intake in the general population [22,23]:

(1) $$\mathrm{DI}\mu g/\mathrm{day}=\frac{UE\left(\mu g/g\right)\times CE\left(\mathrm{mg}/\mathrm{kg}/\mathrm{day}\right)\times M\left(\mathrm{kg}\right)\;}{F_{UE}\times 1000\left(\mathrm{mg}/g\right)}\times \frac{MW_{d}}{MW_{m}}$$

where *UE* is the urinary excretion of benzene metabolite S-PMA or *t, t*-MA in μg/g creatinine. *CE* is the creatinine excretion rate set to be 18 mg/kg/day for women and 23 mg/kg/day for men [16]. *M* is the weight of the subjects. *F_UE_* is the fractional urinary excretion of benzene as S-PMA (0.11%) or *t, t*-MA (3.9%) in humans [24,25]. *MW_d_* and *MW_m_* are the molecular weights of benzene and metabolites. The molecular weights of benzene, S-PMA, and *t, t*-MA were 78.1, 239.3, and 142.1.

### 2.5. Back Calculated Airborne Benzene Levels

Based on the DI values of benzene, we back-calculated airborne benzene levels (BCABL) before applying the following Equation (2). An amount of 10 m^3^ was the default human occupational volume of air inhaled in an 8 h work shift, and the inhalation absorption rate of benzene exposure was 50% [26,27]. Given that in a range of benzene exposure (median: 3.85 mg/m^3^, range: 0.096–64 mg/m^3^), the dose-specific levels of benzene metabolites (µmol/L/ppm benzene) declined with increasing benzene exposure, with a 3-fold reduction in *t, t*-MA, and no decreasing trend for S-PMA [14,28], we introduced a factor *k* (*k* was 3 for *t, t*-MA, and 1 for S-PMA) into the analysis of airborne benzene levels based on *t, t*-MA and S-PMA.

(2) $$\mathrm{BCABL}\;\left(\mathrm{mg}/m^{3}\right)=\frac{\mathrm{DI}}{10m^{3}\times 1000\times 50\%}\times k$$

### 2.6. Statistical Analysis

Statistical analysis was performed with the statistical package SPSS (Version 26.0). Non-detectable levels of airborne benzene were replaced by LOQ/2 (using the 49 control subjects). Airborne benzene and its two metabolites’ values were transformed logarithmically to achieve an approximately normal distribution. A simple linear regression and Pearson’s correlation were used to estimate the correlation coefficients. The Wilcoxon rank-sum test, Mann–Whitney U-test, and Chi-square test were applied to determine statistical differences between the groups. A *p*-value lower than 0.05 was considered to be statistically significant for all tests.

## 3. Results

### 3.1. Summary Statistics

As shown in Table 1, the characteristics of the exposed subjects and control subjects were similar in age, gender, weight, and working duration. The mean age for the benzene-exposed subjects was 34.2 (SD, 10.0) years, and for control subjects it was 32.5 (SD, 6.33) years; 46% of the exposed subjects and 51% of the controls were male. The mean working duration for the exposed subjects was 1.89 years (SD, 1.32). Subjects in the exposed group had higher rates of smoking compared with the unexposed group (23% vs. 8%; *p* = 0.03). Among the exposed subjects, the median measured airborne benzene level was 3.27 mg/m^3^ (though it ranged from 1.10 to 86.2 mg/m^3^). Among the control subjects, the levels of measured airborne benzene were all below the quantitation limit (0.024 mg/m^3^).

The levels of benzene metabolites are summarized in Table 2. Among the exposed subjects, the median levels for S-PMA were 17.0 μg/g creatinine, and for *t, t*-MA, they were 371.4 μg/g creatinine, while, in the control subjects, the two values were 0.38 μg/g creatinine and 68.4 μg/g creatinine, respectively. No significant differences were observed between smokers and non-smokers for the benzene metabolite levels in the exposed subjects (*p* = 0.176 for S-PMA and *p* = 0.100 for *t, t*-MA). Among the control subjects, smokers had higher values of S-PMA (1.31 versus 0.36 μg/g creatinine, *p* = 0.001) and *t, t*-MA (117.2 versus 63.9 μg/g creatinine, *p* = 0.004) than non-smokers.

**Figure 1:**
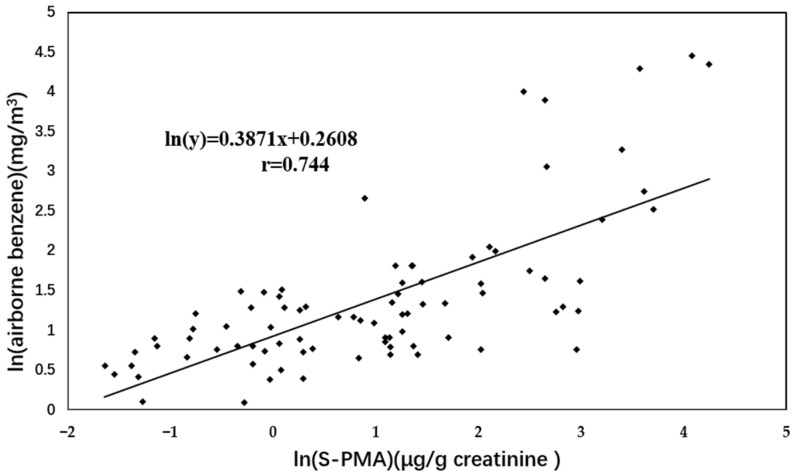
Correlation between natural logarithmic transformed benzene exposure levels (mg/m^3^) and S-PMA values (μg/g creatinine).

**Figure 2:**
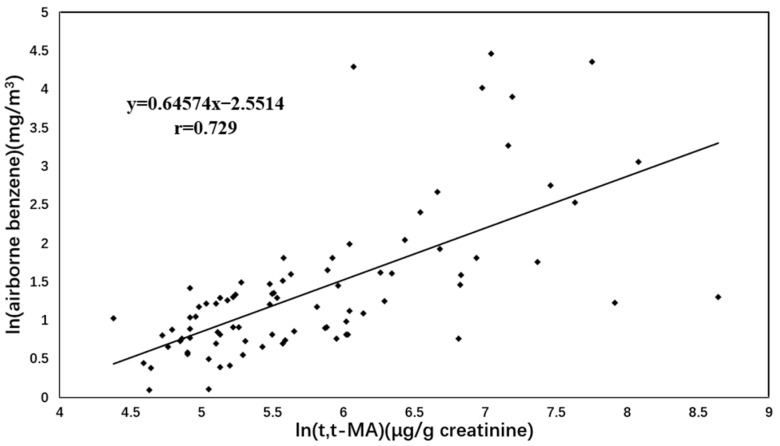
Correlation between natural logarithmic transformed benzene exposure levels (mg/m^3^) and *t, t*-MA values (μg/g creatinine).

As shown in the scatter plots for the measured airborne benzene levels versus S-PMA (Figure 1) and *t, t*-MA (Figure 2), measured airborne benzene concentrations were significantly correlated with S-PMA (r = 0.744, *p* < 0.001) and *t, t*-MA (r = 0.729, *p* < 0.001), respectively. 

### 3.2. Comparison between Measured Airborne Benzene Levels and Back Calculated Airborne Benzene Levels

Table 3 shows the DI values for the benzene of the exposed and control groups. The median DI values among the exposed subjects were 6919.1 μg/day based on urinary S-PMA and 6123.8 μg/day based on urinary *t, t*-MA, while in the control subjects, the two values were 157.3 μg/day and 1330.6 μg/day, respectively. 

BCABL was calculated based on DI using Formula (2). Among the exposed subjects, the median BCABL based on urinary *t, t*-MA was 3.67 mg/m^3^ (range: from 0.87 to 78.6 mg/m^3^), which was very close to the median of the measured airborne benzene level of 3.27 mg/m^3^ (range: from 1.10 to 86.2 mg/m^3^) and there was no significant difference between the BCABL and the measured airborne benzene level (*p* = 0.171). The measured airborne benzene concentrations were significantly correlated with BCABL based on *t, t*-MA (r = 0.729, *p* < 0.001). However, the median BCABL based on urinary S-PMA was 1.39 mg/m^3^ (range: from 0.04 to 106.0 mg/m^3^), which was significantly lower than the measured median airborne benzene level of 3.27 mg/m^3^ (*p* < 0.01). In the controls, the levels of measured airborne benzene were all below the limit of detection (0.024 mg/m^3^) and median BCABLs, which were 0.03 mg/m^3^ based on S-PMA and 0.80 mg/m^3^ based on *t, t*-MA.

The above results indicate that the benzene exposure level was able to be accurately extrapolated by urinary *t, t*-MA. However, the urinary *t, t*-MA concentration might be affected by smoking and dietary habits [29]. In order to further clarify the accuracy of BCABL calculated by *t, t*-MA, we further investigated the effects of smoking habits and dietary habits on BCABL results.

### 3.3. Comparison of BCABL Based on t, t-MA with Respect to Smoking in EXPOSED Subjects

As shown in Table 4, the median levels of BCABL in smokers (3.20 mg/m^3^) and non-smokers (3.85 mg/m^3^) are close to the median measured airborne benzene level (2.49 mg/m^3^ in smokers and 3.36 mg/m^3^ in non-smokers, respectively). Therefore, there are no significant differences between BCABL and the measured airborne benzene levels (all *p* > 0.05). Considering that smoking may exhibit more impact on *t, t*-MA at low levels of benzene exposure, the subjects were categorized according to the median measured airborne benzene level of 3.27 mg/m^3^. For the subjects exposed to ≤3.27 mg/m^3^, the median level of BCABL was 2.58 mg/m^3^ in smokers compared to 2.34 mg/m^3^ in non-smokers, which is close to the median measured airborne benzene level (2.04 mg/m^3^ in smokers, *p* > 0.05; 2.24 mg/m^3^ in non-smokers, *p* > 0.05, respectively). For the subjects exposed to >3.27 mg/m^3^, there are no significant differences between BCABL and the measured airborne benzene level in smokers and non-smokers (all *p* > 0.05). The results indicate that the BCABL based on *t, t*-MA in benzene-exposed subjects was not affected by smoking habits.

### 3.4. Comparison of BCABL Based on t, t-MA with Respect to Dietary Habit in Exposed Subjects

Sorbic acid, a wildly used food preservative, can be metabolized to *t, t*-MA [30]. So, we further analyzed the effect of dietary habits on BCABL. The subjects were categorized according to the median measured airborne benzene level of 3.27 mg/m^3^. As shown in Table 5, there was no significant difference between the BCABL and measured airborne benzene level (all *p* > 0.05) between the subjects who ate preserved food and the subjects who did not eat preserved food. In the group of less than 3.27 mg/m^3^, the median BCABLs was 2.62 mg/m^3^ for the subjects who ate preserved food and 2.51 mg/m^3^ for the subjects who did not eat preserved food, which was close to the median measured airborne benzene level of 2.24 mg/m^3^ and 2.13 mg/ m^3^, respectively. Similar conclusions were found in the group of more than 3.27 mg/m^3^. The median levels of BCABL in subjects who ate preserved food and subjects who did not eat preserved food (4.08 mg/m^3^ and 8.62 mg/m^3^, respectively) were close to the median measured airborne benzene level (4.46 mg/m^3^ and 4.99 mg/m^3^, respectively). There was no significant difference between the BCABL and the measured airborne benzene level (all *p* > 0.05) in subjects who ate preserved food compared to subjects who did not eat preserved food, no matter if the airborne benzene exposure level was below or above 3.27 mg/m^3^.

## 4. Discussion

CYP450 enzymes play an essential role in benzene metabolism. Different metabolic pathways of benzene compete for CYP450. The production of *t, t*-MA requires the secondary oxidation of CYP450 enzymes, while S-PMA is formed by the conjugation of benzene oxide with glutathione, which does not require the secondary oxidation of CYP450 enzymes [31]. For metabolites that require secondary oxidation by CYP450 enzymes, their production rate may decrease with increasing benzene exposure due to the saturation of benzene metabolism [32]. In support of this is the evidence that the dose-specific levels (µmol/L/ppm benzene) of *t, t*-MA decrease about 3-fold when benzene exposure levels increase in the range of 0.096–64 mg/m^3^, with no decreasing trend for S-PMA [28]. Interestingly, considering the effect of the 3-fold-reduced metabolic rate on *t, t*-MA, we found that the *t, t*-MA-based BCBAL (median:3.67 mg/m^3^) was very close to the measured airborne benzene level (median: 3.27 mg/m^3^, *p* = 0.171) of subjects exposed to benzene in the range of 1.10–86.2 mg/m^3^. Furthermore, the stratified analysis did not find the effect of smoking and dietary habit on *t, t*-MA concentration. Our findings suggest that airborne benzene levels can be accurately estimated by BCABL based on *t, t*-MA. As far as we know, this method has been used for the first time to estimate the benzene exposure level for occupational benzene-exposed populations. 

Upon the findings for the S-PMA-based BCBAL examined here, we noted that the S-PMA-based BCBAL in control subjects (range: 2–250 μg/m^3^) was close to the ambient levels of benzene from 2001 to 2016 (0.19–53.90 μg/m^3^) in China [33], and similar to the benzene exposure concentration in the general environment of the United States (0–47.3 μg/m^3^) [34]. However, the S-PMA-based BCBAL in the benzene-exposed subjects (median: 1.39 mg/m^3^) was far from the measured airborne benzene level (median: 3.27 mg/m^3^). One possible interpretation for the above results is that the amount of S-PMA in urine is related to the saturable enzymatic metabolic pathway too. However, the results of previous studies were inconsistent. Melikian et al. [15] found that urinary S-PMA displayed a downward trend of dose-related production. The dose-specific levels (µmol/L/ppm benzene) of S-PMA decreased about 2.7-fold with the increase of benzene exposure levels from a range of 1.1 to 73.5 mg/m^3^. The report of Lung-Cheng Lin et al. [35] showed the same phenomenon of S-PMA production. However, the report of Kim et al. [14] did not show a decreasing trend for S-PMA, with benzene exposure levels increasing in the range of 0.096–64 mg/m^3^. Our results also show that the maximum S-PMA result for the non-smokers of the exposed subjects was very high (1908.7 μg/g creatinine). This may be because the worker was exposed to high concentrations of benzene in the workplace. Another possible reason is that the glutathione S-transferase (GST) genetic polymorphism influenced the excretion levels of S-PMA. A previous study noted that the levels of S-PMA in the subjects with the GSTT1-positive genotype were higher than those with GSTT1-deficient genotypes [35]. In this study, S-PMA is suitable to extrapolate low benzene exposure levels (below 250 μg/m^3^), and the BCABL calculated by S-PMA in the control subjects can be used as a background reference for benzene occupational exposure. However, S-PMA and the BCABL calculated by S-PMA produced a lack of assessment for the benzene exposure of the exposed subjects.

A study has indicated that as a biomarker of benzene exposure, *t, t*-MA might not be able to reliably evaluate the benzene level at very low exposure concentrations (below 1.6 mg/m^3^) [36]. Consistently, we found that the measured airborne benzene concentration of the control group was below the quantitation limit (0.024 mg/m^3^), but BCBAL calculated by *t, t*-MA (median: 0.80 mg/m^3^) was much higher than the quantitation limit. This may be due to the effect of non-benzene sources on *t, t*-MA at low levels of benzene. The background concentration of *t, t*-MA is not only derived from the metabolism of benzene but is also affected by dietary habits and smoking habits. In addition, *t, t*-MA is a metabolite of sorbic acid that can be present in various foodstuffs. The percentage of *t, t*-MA in non-smokers, which was attributed to dietary sorbic acid, was about 10–50% [37]. This study found that the levels of *t, t*-MA in smokers of the control group were significantly higher than those in the non-smoker’s group. Similar results were reported in previous studies [11,38]. Consistently, in the control group of our study, smokers had higher values of *t, t*-MA (117.2 versus 63.9 μg/g creatinine, *p* = 0.004) than non-smokers. Therefore, BCABL, when calculated based on *t, t*-MA, may not accurately reflect benzene exposure for the non-occupational benzene-exposed populations.

## 5. Conclusions

Our study confirmed that the BCABL determined from *t, t*-MA-based DI reflected the actual airborne benzene levels that ranged from 1.10 mg/m^3^ to 86.2 mg/m^3^, and the BCABL from the S-PMA-based DI was more reliable for non-professional benzene exposure. 

## Figures and Tables

**Table 1:** Descriptive parameters of control and exposed subjects.

	Controls (*n* = 49)	Exposure (*n* = 84)	*p*-Value
Age (years)	32.5 ± 6.33	34.2 ± 10.0	0.87 ^a^
Weight (kg)	66.1 ± 9.56	64.8 ± 9.77	0.34 ^a^
Working duration (years)	2.12 ± 1.11	1.89 ± 1.32	0.12 ^a^
Gender [*n* (%)]			0.61 ^b^
Male	25 (51)	39 (46)	
Female	24 (49)	45 (54)	
Current smokers [*n* (%)]			0.03 ^b^
Yes	4 (8)	19 (23)	
No	45 (92)	65 (77)	
Measured airborne benzene (mg/m^3^)			<0.001 ^a^
Median	<0.024	3.27	
Range	<0.024	1.10–86.2	

^a^ Wilcoxon rank-sum test between controls and exposed. ^b^ Chi-square test between controls and exposed.

**Table 2:** Summary statistics for urinary metabolite excretion in control and exposed subjects.

	Exposed			Control		
Variable	Total (*n* = 84)	Smokers (*n* = 19)	Non-Smokers (*n* = 65)	Total (*n* = 49)	Smokers (*n* = 4)	Non-Smokers (*n* = 45)
S-PMA (μg/g creatinine)						
Median	17.0	15.3	20.2	0.38	1.31	0.36
Range	0.72–1908.7	1.26–150.8	0.72–1908.7	0.02–2.34	0.62–2.34	0.02–1.62
*p*-value	<0.001 ^a^	0.176 ^b^			0.001 ^b^	
*t, t*-MA (μg/g creatinine)						
Median	371.4	264.8	404.0	68.4	117.2	63.9
Range	102.1–8331.2	102.1–2773.8	107.4–8331.2	26.1–252.4	41.1–186.6	26.1–252.4
*p*-value	<0.001 ^a^	0.100 ^b^			0.004 ^b^	

A Mann–Whitney U-test *p*-value < 0.05 was considered as statistically significant. ^a^ exposed vs. controls. ^b^ Smokers vs. non-smokers, in exposed subjects or in controls.

**Table 3:** DI ^a^ values of benzene and BCABL ^b^ based on S-PMA and *t, t*-MA in control and exposed subjects.

Group (*n*)	Measured Airborne Benzene (mg/m^3^)	SPMA-Based Calculation		*t, t*-MA-Based Calculation	
		DI (μg/day)	BCABL (mg/m^3^)	DI (μg/day)	BCABL (mg/m^3^)
Controls (49)	<0.024	157.3 (9.62–1259.8)	0.03 (0.002–0.25)	1330.6 (405.1–4536.5)	0.80 (0.24–2.72)
Exposed (84)	3.27 (1.10–86.2)	6919.1 (211.0–530,199.6)	1.39 ^c^ (0.04–106.0)	6123.8 (1447.0–131,036.1)	3.67 ^c^ (0.87–78.6)

^a^ DI—daily intake. ^b^ BCABL—back calculated airborne benzene levels. ^c^ *p*-value between airborne benzene and BCABL (based on S-PMA or *t, t*-MA): *p* < 0.01 for S-PMA and *p* = 0.171 for *t, t*-MA.

**Table 4:** Summary of airborne benzene and BCABL based on *t, t*-MA in smokers and non-smokers.

Subgroup (*n*)	Airborne Benzene (mg/m^3^)	BCABL (mg/m^3^)	*p*-Value
Smokers (19)	2.49 (1.47–15.6)	3.20 (1.40–34.5)	0.091 ^a^
≤3.27 mg/m^3^ (12)	2.04 (1.47–3.08)	2.58 (1.39–7.58)	0.200 ^a^
>3.27 mg/m^3^ (7)	3.85 (3.63–15.6)	5.03 (2.96–34.5)	0.710 ^a^
Non-Smokers (65)	3.36 (1.10–86.2)	3.85 (0.87–78.6)	0.483 ^a^
≤3.27 mg/m^3^ (30)	2.24 (1.10–3.22)	2.34 (0.87–19.8)	0.525 ^a^
>3.27 mg/m^3^ (35)	5.03 (3.32–86.2)	6.52 (1.01–78.6)	0.883 ^a^

^a^*p*-value between airborne benzene and BCABL (based on *t, t*-MA).

**Table 5:** Summary of airborne benzene and BCABL based on *t, t*-MA considering dietary habit.

Subgroup (*n*)	Airborne Benzene (mg/m^3^)	BCABL (mg/m^3^)	*p*-Value
Eat preserved food (30)	3.57 (1.73–73.3)	3.39 (0.98–78.6)	0.836 ^a^
≤3.27 mg/m^3^ (11)	2.24 (1.73–2.98)	2.62 (0.98–11.1)	0.401 ^a^
>3.27 mg/m^3^ (19)	4.46 (3.32–73.3)	4.08 (1.01–78.6)	0.246 ^a^
Not eat preserved food (54)	2.84 (1.10–86.2)	3.87 (0.87–57.0)	0.073 ^a^
≤3.27 mg/m^3^ (31)	2.13 (1.10–3.22)	2.51 (0.87–19.8)	0.120 ^a^
>3.27 mg/m^3^ (23)	4.99 (3.43–86.2)	8.62 (2.68–57.0)	0.089 ^a^

^a^*p*-value between airborne benzene and BCABL (based on *t, t*-MA).

## Data Availability

The data presented in this study are available on request from the corresponding author. The data are not publicly available due to the privacy of subjects.
